# 2429. Epidemiology and Risk Factors for Vascular Access-associated Infections in Japanese Hemodialysis Patients

**DOI:** 10.1093/ofid/ofad500.2048

**Published:** 2023-11-27

**Authors:** Keita Morikane

**Affiliations:** Yamagata University Hospital, Yamagata, Yamagata, Japan

## Abstract

**Background:**

Surveillance of vascular access-associated infections (VAIs) in patients on hemodialysis has been well established in the United States, but not so in other countries including Japan. Our group established a voluntary VAI surveillance scheme, Dialysis Surveillance Network Japan (DSNJ), which is in operation for over 10 years. The purpose of this study is to describe the epidemiology and identify risk factors for VAI in patients on hemodialysis in Japan.

**Methods:**

Data collected through DSNJ from January 2008 to December 2021 were used. Incidence of VAI was calculated by the number of infection per 1,000 dialysis sessions. Potential risk factors analyzed in this study included type of access, diabetes, indication of catheter use and seasonality

**Results:**

During the study period, 4,919,834 dialysis sessions were surveyed at 52 hospitals and 1,146 VAI were observed, with an overall incidence of 0.23 per 1,000 sessions. The incidence declined over time. The incidences by the type of access were 0.05 (203 VAI in 4,285,758 dialysis sessions) for arteriovenous fistula (AVF), 0.11 (17/157,754) for superficialization of brachial artery (SBA), 0.53 (127/240,289) for arteriovenous graft (AVG), 1.33 (219/164,195) for cuffed catheter (CC) and 8.07 (580/71,838) for non-cuffed catheter (NCC). NCC had significantly higher risk for VAI than any other type of access. The most frequently accountable pathogen was methicillin-susceptible *Staphylococcus aureus*. The risk of diabetes for VAI was not significant in patients with either NCC or CC (NCC: RR 1.18, 95%CI: 0.98-1.43, CC: RR0.86, 95%CI: 0.64-1.16). NCC inserted at the femoral site had significantly higher risk of VAI compared to that at internal jugular site (RR 1.47, 95%CI: 1.21-1.78). This was also true for CC (RR:2.41, 95%CI: 1.34-4.34). NCC used at the induction of hemodialysis was more likely to be complicated with VAI than NCC used as a temporary substitute (RR 1.50, 95%CI: 1.22-1.84). Fifty-five percent (634/1,146) of VAI occurred in summer (May to October), indicating a seasonal variation in the incidence of VAI.

**Conclusion:**

Our surveillance scheme revealed epidemiology and risk factors for VAI in Japanese hemodialysis patients. These findings might lead to better control VAIs in patients on hemodialysis.

**Disclosures:**

**All Authors**: No reported disclosures

